# Circulating MicroRNAs in gynecological malignancies: from detection to prediction

**DOI:** 10.1186/2162-3619-3-14

**Published:** 2014-05-20

**Authors:** Ya-Nan Zhao, Guan-Sheng Chen, Shun-Jia Hong

**Affiliations:** 1 Guangdong Provincial Key Laboratory of Malignant Tumor Epigenetics and Gene Regulation, Sun Yat-sen Memorial Hospital, Guangzhou 510120, China; 2 Department of Obstetrics & Gynecology, Sun Yat-sen Memorial Hospital, Sun Yat-sen University, Guangzhou 510120, China

## Abstract

MicroRNAs (miRNAs) have been demonstrated to play critical roles in many physiological and pathophysiological processes. The presence of altered miRNA profiles in human body fluids has been reported for a number of diseases including gynecological malignancies. In this review, we summarized the current progresses of circulating miRNAs associated with malignancies in gynecology, with an emphasizing on the circulating miRNAs as diagnostic and prognostic biomarkers in ovarian cancer, endometrial carcinoma and cervical cancer.

## Introduction

Cancer has long been one of the major causes of death worldwide. Along with its increasing incidence and mortality, enormous efforts have been made on etiology, pathophysiology, diagnosis and therapeutics of multiple malignancies, including gynecological neoplasms. Ovarian cancer, together with endometrial cancer and cervical carcinoma, are the three most common gynecological malignant tumors. They contributed more than 2/3 of new cases of cancer in female genital system every year. And, ovarian cancer, ranked the fifths of all female malignancies, causing 14,270 deaths in US per year [1]. It is the highest in gynecologic tumors.

Current diagnostic methods for cancer, especially malignancies of female reproductive tract, mainly depend on typical symptoms and screening tests, such as pelvic examination, transvaginal ultrasound, and tumor biomarkers. However, many tumors are asymptomatic at early stage, like ovarian cancer. And on the other side, examinations and biomarkers are not yet sensitive or sufficiently specific for tumor detection. Consequently, finding reliable methods/biomarkers for early diagnosis of cancer remains a significant clinical challenge.

MicroRNAs (miRNAs), a class of small RNAs approximately 18–22 nucleotides in length, were initially discovered in 1993. The regulatory function of miRNAs was proven to be inducing either translation suppression or degradation of RNA. Investigations have suggested that miRNAs are related to a lot of physiological processes [2,3], and their dysregulation can lead to the occurrence and progression of diseases. Researchers found that miRNAs might be involved in the pathogenesis of a variety of human cancers, such as lung cancer [4], prostate cancer [5], colorectal tumor [6] and leukemia [7,8], as either oncogenes or onco-suppressors.

Emerging evidences of miRNAs in circulation and in body fluids, such as serum [9], human whole saliva [10], and urine [11], have initiated a new era of disease research. Circulatory miRNAs are reported mainly from monocytes, plasma, and exosomes [9,12], and resistant to degradation by RNase enzyme thus stable in the blood [13]. A lot of profiling studies have linked miRNAs to possible biomarkers for disorders, both diagnostic and prognostic. Consequently, the potential role of miRNAs in clinical practice lies in not only the early diagnosis and classification of tumors, but also the identification of poorly differentiated malignancies and the prediction of survival and response to treatment.

For tumors of human female reproductive tract, investigations have revealed the differential expression of miRNAs in tissue as well as blood/serum samples. These findings had been further linked to recognition and monitoring of tumors. Here in this review we highlighted the new progresses on the role of circulating miRNAs associated with malignancies in gynecology.

### Ovarian cancer and circulating MiRNAs

Ovarian cancer is one of the deadliest gynecologic malignancies worldwide, and epithelial ovarian cancer (EOC) is the most common pathological type of ovarian cancer. Carbohydrate antigen-125 (CA-125), and Human Epididymis Protein 4 (HE4), are the most widely used biomarkers for EOC diagnosis, of which sensitivity and specificity are not quite satisfied. About 70% of patients are diagnosed in advanced clinical stages of the disease due to lack of effective screening methods [14]. Therefore, finding reliable biomarkers for the early diagnosis of ovarian cancer remains a crucial topic.

Proven to be stable and conservative in circulation, a variety of miRNAs were revealed differently expressed in ovarian carcinomas and in disease specific conditions. Advances in applications of miRNAs as diagnostic and prognostic biomarkers have also caught attentions of scientists.

Taylor *et al.* isolated circulating tumor-derived exosomes from sera of patients diagnosed with ovarian serous papillary adenocarcinoma, and for the first time reported that eight circulating miRNAs were differentially expressed between ovarian cancer patients and benign controls [13]. This investigation, followed by several profiling studies [15-19], raised the possibility that miRNAs could potentially be used as surrogate diagnostic markers for biopsy. Table 1 summarized the differential expression miRNAs in profiling studies. As revealed recently, expression level of miR-26a in plasma was reported to be able to distinguish EOC patients from healthy controls [20]. And interestingly, the results of Resnick’s investigation showed that serum levels of miRNAs were not consistent with serum CA-125 levels, the present marker of ovarian tumors [15]. They found miR-21, 92 and 93 were significantly over-expressed in patients with normal CA-125, highlighting the prospect of circulating miRNAs as non-invasive biomarkers of cancer detection.

**Table 1:** Differential expressed circulating microRNAs of ovarian cancer in profiling studies

**Dysregulated microRNAs**		**Associations**	**Histology**	**Sources**	**Methods**	**References**
**Up-regulated**	**Down-regulated**					
miR-21, 29a, 92, 93, 126	miR-127, 155, 99b		ovarian cancer	serum, patient vs. normal control	microarray,qRT-PCR	Resnick, 2009 [14]
95 miRNAs	88 miRNAs, 4 validated by qRT-PCR: miR-132, 26a, let-7b, miR-145		serous ovarian cancer	serum, patient vs. healthy control	microarray,qRT-PCR	Chung, 2013 [15]
miR-205, 643, 200a, 184, 9, 21*, let-7f-2*, miR-483-5p, 616*, 34a*, 501-5p, 141	let-7a, 7d*, 7e*, 106b*, 149, 193b*, 362-3p, 363*, 422a, 449a, 770-5p, 98, 15b*, let-7f, miR-221, 450a, 570, 573	lower let-7f vs. poor prognosis	epithelial ovarian cancer	plasma, patient vs. healthy control	TaqMan Low Density Array, qRT-PCR	Zheng, 2013 [18]
miR-1274a, 30b, 30c, 625-3p, 720;			serous epithelial ovarian cancer	plasma, OC^#^ patient vs. healthy volunteer	TaqMan Open Array	
miR-106b, 126, 150, 17, 20a, 92a;				plasma, OC^#^ patient vs. benign neoplasm		Shapira, 2014 [16]
MiR-139-5p, 142-3p, 484, 486, 660				benign neoplasm vs. healthy volunteer		
miR-21, 191, 16, 15b, 1977, 1979, 1973, 1974, 4284, 195;			EAOC^##^: endometrioidand/or clear cell adenocarcinoma	plasma, EAOC patients vs. healthy controls	RT-qPCR	Suryawanshi, 2013 [17]
miR-362-5p, 1274a, 21, 766, 1975, 1308, 191, 744, 376a, 1246;				plasma, EAOC patients vs. endometriosis patients		
miR-16, 195, 191, 1974, 4284, 15b, 1978, 1979, 362-5p, 1973				endometriosis patients vs. healthy controls		

^#^OC: ovarian cancer, ^##^EAOC: endometriosis-associated ovarian cancer.

On the basis of expression profiling, researchers further tried to analyze the utility of circulating miRNAs in diagnosing ovarian cancers. Kan *et al.*[21] reported that the expressions of miR-200a, miR-200b and 200c were significantly up-regulated in sera of patients diagnosed with serous epithelial ovarian cancer (n = 28). They founded an efficient predictive model with normalized miR-200b and miR-200c (ROC-AUC = 0.784). Although the sample size of this study was limited, it lighted up the chance of using serum miRNAs as promising discriminating tools of ovarian cancer. In a larger sample analysis (360 epithelial ovarian cancer patients and 200 healthy controls), Zheng [19] and coworkers found higher miR-205 and lower let-7f expression in plasma of cases than that of controls. Furthermore, the levels of miR-205 and let-7f together provided high diagnostic accuracy for ovarian cancer patients, especially for stage I disease. The combination of these two miRNAs, together with CA-125 further improved the accuracy of detection, suggesting a good possibility in improving the current deficiency of early ovarian cancer diagnosis.

Despite the potential role of circulating miRNAs in distinguishing ovarian cancers from healthy controls and benign tumors, the expression levels of some miRNAs have been revealed to be associated with some clinicopathological features of cancer. The concentration of miR-221 in sera of patients with epithelial ovarian cancer (EOC) was higher than that of healthy controls [22]. Furthermore, the expression level of serum miR-221 was significantly associated with International Federation of Gynecology and Obstetrics (FIGO) stage and tumor grade. And, through multivariate analysis of overall survival, high serum miR-221 expression was confirmed as an independent unfavorable prognostic factor in EOC. Guo *et al.*[23] found a significant correlation between miR-92 expression and regional lymph node involvement and clinical stage of the tumor, while there was no significant association between expression of miR-92 and age. This suggested the possibility of up-regulated miR-92 levels as a prognostic biomarker. Increased serum miR-21 expression was also proved to be correlated with advanced FIGO stage, high tumor grade, and shortened overall survival [24]. And in Zheng’s study, they raised the correlation between lower let-7f and poor prognosis in EOC patients, indicating let-7f may be predictive of ovarian cancer prognosis [19]. In addition, Shapira *et al.*[17] analyzed the follow-up outcome and pre-surgery plasma miRNA levels of women diagnosed with ovarian cancer. Significant differences in 5 miRNA expressions were found in women who had short overall survival (SOS) compared to women with long overall survival (LOS). And after correction for multiple testing, level of miR-1290 was confirmed significantly different between the two groups (*adj.P*-value = 0.05, AUC = 0.87), giving a great chance of serum miRNAs as monitoring and prognostic biomarkers.

### Circulating MicroRNA signatures in endometrial carcinoma

Endometrial carcinomas is one of the most common malignant tumors of the female reproductive tract, and approximately 80% of endometrial cancers are of endometrioid histology [25]. Diagnosed mostly in early clinical stages, again despite a great improvement in endometrial cancer treatment and diagnosis, advanced stages of the disease are still difficult to manage with the 5-year survival rate of ~10–29% [26]. Highly sensitive and specific molecular prognostic biomarkers are required to better predict the outcome of endometrioid endometrial cancer (EEC). Recent study identified that miR-194 has potential to serve as prognostic biomarker for endometrial cancer [27]. However, circulating miRNAs involved in ECC were not analyzed as much as in ovarian cancers.

Tan *et al.* tried to use the serum level of miR-155 to differentiated EEC from healthy controls, and other histological types of endometrial carcinomas [28]. They found that the increase of miR-155 expression was associated with cancer stage, and lymph node metastasis (LNM). The potential of circulating miRNAs serving as diagnostic and prognostic markers was then confirmed by other researchers. Torres and coworkers [29] found that the expression of miR-99a, miR-100 and miR-199b was up-regulated in plasma of EEC patients, and a combination of miR-99a and miR-199b was more accurate in distinguishing EEC disease when compared with single miRNAs. Another genome-wide serum miRNA expression profile identified a combination of four serum miRNAs (miR-222, miR-223, miR-186 and miR-204) as a fingerprint for EEC detection. The ROC-AUC was 0.927, which was markedly higher than that of CA-125 (0.673), showing a better accuracy.

Other investigations of miRNA profiling in EEC have tried to make associations between circulating miRNAs and the clinicopathological characteristics [30,31]. Expression of a number of miRNAs was showed to have links to signatures like FIGO stage, grade, relapse and nodal metastases, while the relations were mostly linked to miRNAs expression in tissue samples.

### Circulating MiRNA studies in cervical cancer

Cervical cancer is a primary cancer of the uterine cervix in female whose development is a multistep process initiated by persistent infection with high-risk human papillomavirus. Although the mortality of cervical cancer was decreasing in area with advanced healthcare and prevention system, the incidence is still high worldwide. Thanks to the effective screening methods for cervical cancer, studies focused on extracellular miRNAs and cervical cancer were limited. In our recent work, we demonstrated that the circulating miRNAs, miR-646, miR-141* and miR-542-3p were differentially expressed in the serum of cervical squamous cell carcinoma (SCC) patients before and after surgery, thus could potentially serve as non-invasive biomarkers and post-therapeutic monitors for cervical SCC [32].

Researchers investigated the expression of miR-218 in the sera from cervical cancer patients and its relationships with clinicopathological characteristics [33]. MiR-218 was reduced significantly in the sera of cervical cancer patients. Moreover, decreased miR-218 was reported to be associated with later stages, cervical adenocarcinoma, and lymphatic node metastasis. And in another research, Zhao *et al.*[34] found that the expression levels of miR-20a and miR-203 were both significantly higher in cervical cancer patients compared with healthy controls. Patients with LNM tended to have overexpression of miR-20a, but down-regulation of miR-203. The authors took further study to analyze the efficiency of using miR-20a to differentiate LNM (+) patients from LNM (−) patients. The value of the area under the receiver-operating curve (AUC) was 0.734 ± 0.058, the sensitivity and specificity of serum miR-20a were 75% and 72.5%, respectively, and the cut-off point was 3.0. These results suggested that the circulating miR-20a may be a potential biomarker for detecting the lymph node status of cervical cancer patients.

Since LNM is one of the crucial clinicopahtological features of cervical SCC, more than one research focused on the detection of LNM. Chen *et al.*[35] examined the expression levels of miRNAs that dysregulated between LNM (+) and LNM (−) SCC samples, both in tissue samples and in sera of patients and healthy controls. They identified 6 serum miRNAs that can predict LNM in cervical SCC patients: miR-1246, miR-20a, miR-2392, miR-3147, miR-3162-5p and miR-4484. Furthermore, they analyzed the prediction value of LNM using comprehensive set of these serum microRNAs. The ROC-AUC reached to 0.932. As shown in their results, the predictive value of the serum miRNAs was inferior to that in tissue, but far superior to serum SCC antigen (SCC-Ag) analysis, suggesting that serum miRNAs are potential novel predictors of LNM with clinical value in early-stage cervical SCC.

### Future perspectives

Circulating miRNAs have been proposed as diagnostic and prognostic biomarkers for gynecological malignancies, including ovarian cancer, endometrial carcinoma and cervical cancer. There exists a high level of inconsistency across present studies as many results have been reported only within one or a few times so far. And there will be a long way to go before we can really use these findings in clinical work. In addition, the production and metabolic pathways of circulating miRNAs, as well as their function remain unclear to date and need further investigation.

## Competing interests

The authors declare that they have no competing interests.

## Authors’ contributions

YN Zhao, and SJ Hong participated in review design; YN Zhao, and GS Chen performed literature search; and YN Zhao, and SJ Hong wrote or contributed to the writing of the manuscript. All authors read and approved the final manuscript.

## References

[B1] Siegel R Ma J Zou Z Jemal A Cancer statistics, 2014 CA Cancer J Clin 2014 64 9 29 10.3322/caac.21208 24399786

[B2] Bartel DP MicroRNAs: genomics, biogenesis, mechanism, and function Cell 2004 116 281 297 10.1016/S0092-8674(04)00045-5 14744438

[B3] Zhang H Chen Z Wang X Huang Z He Z Chen Y Long non-coding RNA: a new player in cancer J Hematol Oncol 2013 6 37 10.1186/1756-8722-6-37 23725405 PMC3693878

[B4] Hayashita Y Osada H Tatematsu Y Yamada H Yanagisawa K Tomida S Yatabe Y Kawahara K Sekido Y Takahashi T A polycistronic microRNA cluster, miR-17-92, is overexpressed in human lung cancers and enhances cell proliferation Cancer Res 2005 65 9628 9632 10.1158/0008-5472.CAN-05-2352 16266980

[B5] Hassan O Ahmad A Sethi S Sarkar FH Recent updates on the role of microRNAs in prostate cancer J Hematol Oncol 2012 5 9 10.1186/1756-8722-5-9 22417299 PMC3313897

[B6] Akao Y Nakagawa Y Hirata I Iio A Itoh T Kojima K Nakashima R Kitade Y Naoe T Role of anti-oncomirs miR-143 and −145 in human colorectal tumors Cancer Gene Ther 2010 17 398 408 10.1038/cgt.2009.88 20094072

[B7] Sun YM Lin KY Chen YQ Diverse functions of miR-125 family in different cell contexts J Hematol Oncol 2013 6 6 10.1186/1756-8722-6-6 23321005 PMC3566921

[B8] Gimenes-Teixeira HL Lucena-Araujo AR Dos SG Zanette DL Scheucher PS Oliveira LC Dalmazzo LF Silva-Junior WA Falcao RP Rego EM Increased expression of miR-221 is associated with shorter overall survival in T-cell acute lymphoid leukemia Exp Hematol Oncol 2013 2 10 10.1186/2162-3619-2-10 23566596 PMC3637292

[B9] Mitchell PS Parkin RK Kroh EM Fritz BR Wyman SK Pogosova-Agadjanyan EL Peterson A Noteboom J O’Briant KC Allen A Lin DW Urban N Drescher CW Knudsen BS Stirewalt DL Gentleman R Vessella RL Nelson PS Martin DB Tewari M Circulating microRNAs as stable blood-based markers for cancer detection Proc Natl Acad Sci U S A 2008 105 10513 10518 10.1073/pnas.0804549105 18663219 PMC2492472

[B10] Patel RS Jakymiw A Yao B Pauley BA Carcamo WC Katz J Cheng JQ Chan EK High resolution of microRNA signatures in human whole saliva Arch Oral Biol 2011 56 1506 1513 10.1016/j.archoralbio.2011.05.015 21704302 PMC3189266

[B11] Wang G Chan ES Kwan BC Li PK Yip SK Szeto CC Ng CF Expression of microRNAs in the urine of patients with bladder cancer Clin Genitourin Cancer 2012 10 106 113 10.1016/j.clgc.2012.01.001 22386240

[B12] Hausler SF Keller A Chandran PA Ziegler K Zipp K Heuer S Krockenberger M Engel JB Honig A Scheffler M Dietl J Wischhusen J Whole blood-derived miRNA profiles as potential new tools for ovarian cancer screening Br J Cancer 2010 103 693 700 10.1038/sj.bjc.6605833 20683447 PMC2938264

[B13] Taylor DD Gercel-Taylor C MicroRNA signatures of tumor-derived exosomes as diagnostic biomarkers of ovarian cancer Gynecol Oncol 2008 110 13 21 10.1016/j.ygyno.2008.04.033 18589210

[B14] Cannistra SA Cancer of the ovary N Engl J Med 2004 351 2519 2529 10.1056/NEJMra041842 15590954

[B15] Resnick KE Alder H Hagan JP Richardson DL Croce CM Cohn DE The detection of differentially expressed microRNAs from the serum of ovarian cancer patients using a novel real-time PCR platform Gynecol Oncol 2009 112 55 59 10.1016/j.ygyno.2008.08.036 18954897

[B16] Chung YW Bae HS Song JY Lee JK Lee NW Kim T Lee KW Detection of microRNA as novel biomarkers of epithelial ovarian cancer from the serum of ovarian cancer patients Int J Gynecol Cancer 2013 23 673 679 10.1097/IGC.0b013e31828c166d 23542579

[B17] Shapira I Oswald M Lovecchio J Khalili H Menzin A Whyte J Dos SL Liang S Bhuiya T Keogh M Mason C Sultan K Budman D Gregersen PK Lee AT Circulating biomarkers for detection of ovarian cancer and predicting cancer outcomes Br J Cancer 2014 110 976 983 10.1038/bjc.2013.795 24366298 PMC3929876

[B18] Suryawanshi S Vlad AM Lin HM Mantia-Smaldone G Laskey R Lee M Lin Y Donnellan N Klein-Patel M Lee T Mansuria S Elishaev E Budiu R Edwards RP Huang X Plasma microRNAs as novel biomarkers for endometriosis and endometriosis-associated ovarian cancer Clin Cancer Res 2013 19 1213 1224 10.1158/1078-0432.CCR-12-2726 23362326 PMC3596045

[B19] Zheng H Zhang L Zhao Y Yang D Song F Wen Y Hao Q Hu Z Zhang W Chen K Plasma miRNAs as diagnostic and prognostic biomarkers for ovarian cancer PLoS One 2013 8 e77853 10.1371/journal.pone.0077853 24223734 PMC3815222

[B20] Shen W Song M Liu J Qiu G Li T Hu Y Liu H MiR-26a promotes ovarian cancer proliferation and tumorigenesis PLoS One 2014 9 e86871 10.1371/journal.pone.0086871 24466274 PMC3899311

[B21] Kan CW Hahn MA Gard GB Maidens J Huh JY Marsh DJ Howell VM Elevated levels of circulating microRNA-200 family members correlate with serous epithelial ovarian cancer BMC Cancer 2012 12 627 10.1186/1471-2407-12-627 23272653 PMC3542279

[B22] Hong F Li Y Xu Y Zhu L Prognostic significance of serum microRNA-221 expression in human epithelial ovarian cancer J Int Med Res 2013 41 64 71 10.1177/0300060513475759 23569131

[B23] Guo F Tian J Lin Y Jin Y Wang L Cui M Serum microRNA-92 expression in patients with ovarian epithelial carcinoma J Int Med Res 2013 41 1456 1461 10.1177/0300060513487652 23963852

[B24] Xu YZ Xi QH Ge WL Zhang XQ Identification of serum microrna-21 as a biomarker for early detection and prognosis in human epithelial ovarian cancer Asian Pac J Cancer Prev 2013 14 1057 1060 10.7314/APJCP.2013.14.2.1057 23621186

[B25] Amant F Moerman P Neven P Timmerman D van Limbergen E Vergote I Endometrial cancer Lancet 2005 366 491 505 10.1016/S0140-6736(05)67063-8 16084259

[B26] Bansal N Yendluri V Wenham RM The molecular biology of endometrial cancers and the implications for pathogenesis, classification, and targeted therapies Cancer Control 2009 16 8 13 19078924 10.1177/107327480901600102

[B27] Zhai H Karaayvaz M Dong P Sakuragi N Ju J Prognostic significance of miR-194 in endometrial cancer Biomark Res 2013 1 12 10.1186/2050-7771-1-12 24040515 PMC3772652

[B28] Tan ZQ Liu FX Tang HL Su Q [Expression and its clinical significance of hsa-miR-155 in serum of endometrial cancer] Zhonghua Fu Chan Ke Za Zhi 2010 45 772 774 21176560

[B29] Torres A Torres K Pesci A Ceccaroni M Paszkowski T Cassandrini P Zamboni G Maciejewski R Deregulation of miR-100, miR-99a and miR-199b in tissues and plasma coexists with increased expression of mTOR kinase in endometrioid endometrial carcinoma BMC Cancer 2012 12 369 10.1186/1471-2407-12-369 22920721 PMC3495850

[B30] Torres A Torres K Pesci A Ceccaroni M Paszkowski T Cassandrini P Zamboni G Maciejewski R Diagnostic and prognostic significance of miRNA signatures in tissues and plasma of endometrioid endometrial carcinoma patients Int J Cancer 2013 132 1633 1645 10.1002/ijc.27840 22987275

[B31] Tsukamoto O Miura K Mishima H Abe S Kaneuchi M Higashijima A Miura S Kinoshita A Yoshiura KI Masuzaki H Identification of endometrioid endometrial carcinoma-associated microRNAs in tissue and plasma Gynecol Oncol 2014 132 715 721 10.1016/j.ygyno.2014.01.029 24491411

[B32] Wang WT Zhao YN Yan JX Weng MY Wang Y Chen YQ Hong SJ Differentially expressed microRNAs in the serum of cervical squamous cell carcinoma patients before and after surgery J Hematol Oncol 2014 7 6 10.1186/1756-8722-7-6 24405714 PMC3892020

[B33] Yu J Wang Y Dong R Huang X Ding S Qiu H Circulating microRNA-218 was reduced in cervical cancer and correlated with tumor invasion J Cancer Res Clin Oncol 2012 138 671 674 10.1007/s00432-012-1147-9 22237456 PMC11824354

[B34] Zhao S Yao D Chen J Ding N Circulating miRNA-20a and miRNA-203 for screening lymph node metastasis in early stage cervical cancer Genet Test Mol Biomarkers 2013 17 631 636 10.1089/gtmb.2013.0085 23819812

[B35] Chen J Yao D Li Y Chen H He C Ding N Lu Y Ou T Zhao S Li L Long F Serum microRNA expression levels can predict lymph node metastasis in patients with early-stage cervical squamous cell carcinoma Int J Mol Med 2013 32 557 567 23799609 10.3892/ijmm.2013.1424 PMC3782554

